# Supplementary material for: A novel class of sulfur-containing aminolipids widespread in marine roseobacters
Source: ISME J. 2021 Mar 9;15(8):2440–53. doi: 10.1038/s41396-021-00933-x (PMC8319176; doi:10.1038/s41396-021-00933-x)
Supplement: Supplementary file 1 — supplementary table 1 [file 41396_2021_933_MOESM1_ESM.docx]

**Table S1,** Primers used for molecular cloning and PCR amplification

| **Locus Tag** | **Use** | **Forward primer (5’-3’)** | **Reverse primer (5’-3’)** |
| --- | --- | --- | --- |
| pK18*mobsacB* | Linearized vector | TGGCACTGGCCGTCGTTT | TACCGAGCTCGAATTCGTAATCATG |
| SPO2471 | Upstream region A | AACTTAATCGCCTTGCAGCACAATATCGCCACCGCATTCA | TTCTGGACCAGTTGCGTGAGGTCAGCCGGTCGAGATCCTC |
| P34S-Gm | Amplification of Gm cassette | GAGGATCTCGACCGGCTGACCTCACGCAACTGGTCCAGAA | TCATCAGCATGTAATCGGGGCGGCGTTGTGACAATTTACC |
| SPO2471 | Downstream region B | GGTAAATTGTCACAACGCCGCCCCGATTACATGCTGATGA | CTTTCTACGTGTTCCGCTTCCCGCCCATAACGACGATCTG |
| SPO2471 | Confirmation of mutant | CGCATGCCGTCGATTTCATCAT | AGGTCTACCTAGCCTCTTGCGC |
|  |  |  |  |
| SPO0716 | Amplification of *aacC1* promoter | CCACCGCGGTGGCGGCCGCTGTCGACTCTAGAGGATCC | TTTCCACCACCGTTGCTGCTCCATAACATC |
| SPO0716 | Amplification for complementation | AGCAGCAACGGTGGTGGAAACCGCGCAG | CGAATTCCTGCAGCCCGGGGCTAGCCGCCAAGCGACAG |
|  |  |  |  |
| PGA1_c01210 | Amplification of *aacC1* promoter | CCACCGCGGTGGCGGCCGCTGTCGACTCTAGAGGATCC | TATCCGCCAACGTTGCTGCTCCATAACATC |
| PGA1_c01210 | Amplification for complementation | AGCAGCAACGTTGGCGGATACTGCGCATG | CGAATTCCTGCAGCCCGGGGTCAGCTGTTTTCGCCGAG |
