# Supplementary material for: A novel class of sulfur-containing aminolipids widespread in marine roseobacters
Source: ISME J. 2021 Mar 9;15(8):2440–53. doi: 10.1038/s41396-021-00933-x (PMC8319176; doi:10.1038/s41396-021-00933-x)
Supplement: Supplementary file 2 — supplementary figure 1 [file 41396_2021_933_MOESM2_ESM.docx]

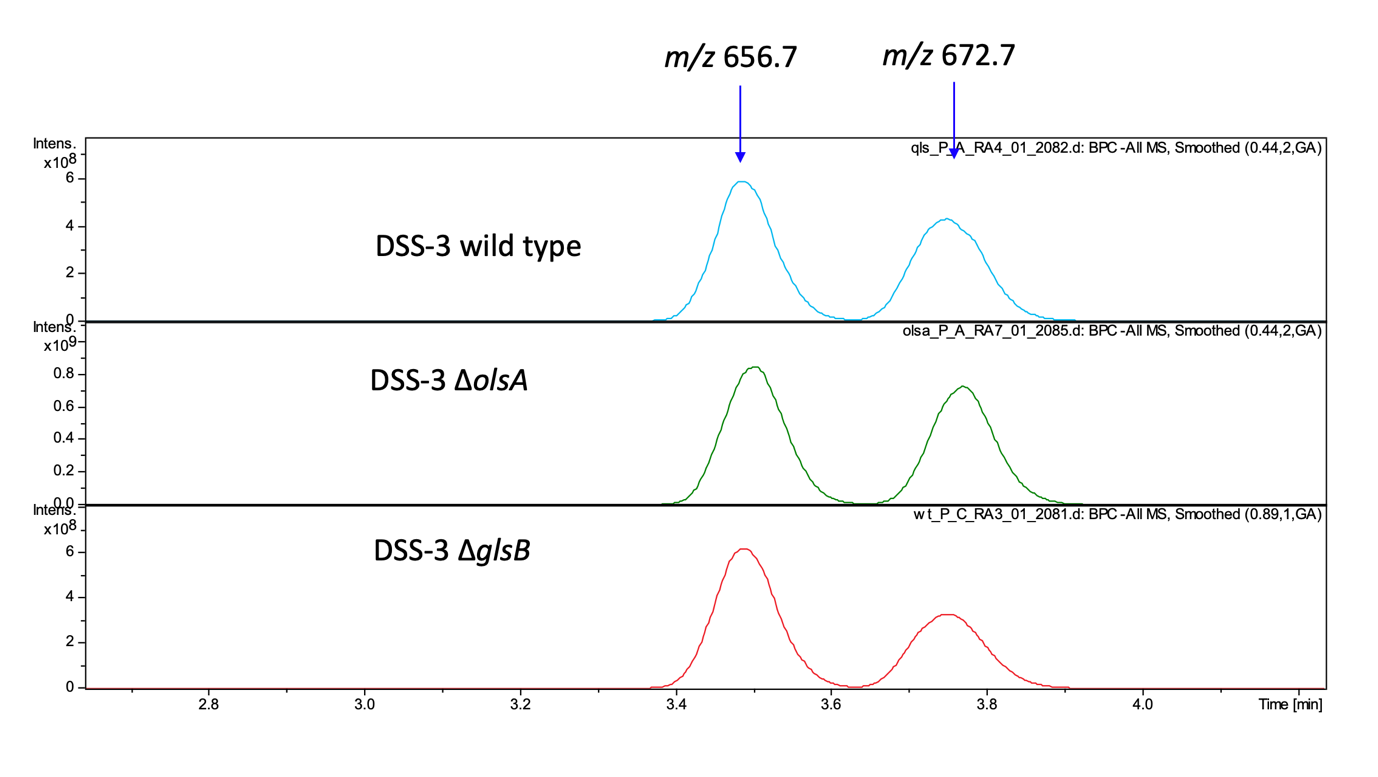


**Figure S1**, Sulfur-containing aminolipid (SAL) production in *Ruegeria pomeroyi* DSS-3 is not affected in the *olsA* mutant or the *glsB* mutant. The blue arrows point to the two major SAL species eluted at 3.4-4 min, with a *m/z* of 656.7 and 672.7 respectively. Cells were cultivated in ½ YTSS medium. Ions were collected in the negative (-ve) ionisation mode using electrospray ionisation (ESI) using an amaZon SL ion trap MS (Bruker).
