# Supplementary material for: A novel class of sulfur-containing aminolipids widespread in marine roseobacters
Source: ISME J. 2021 Mar 9;15(8):2440–53. doi: 10.1038/s41396-021-00933-x (PMC8319176; doi:10.1038/s41396-021-00933-x)
Supplement: Supplementary file 3 — supplementary figure 2 [file 41396_2021_933_MOESM3_ESM.docx]

**a)**


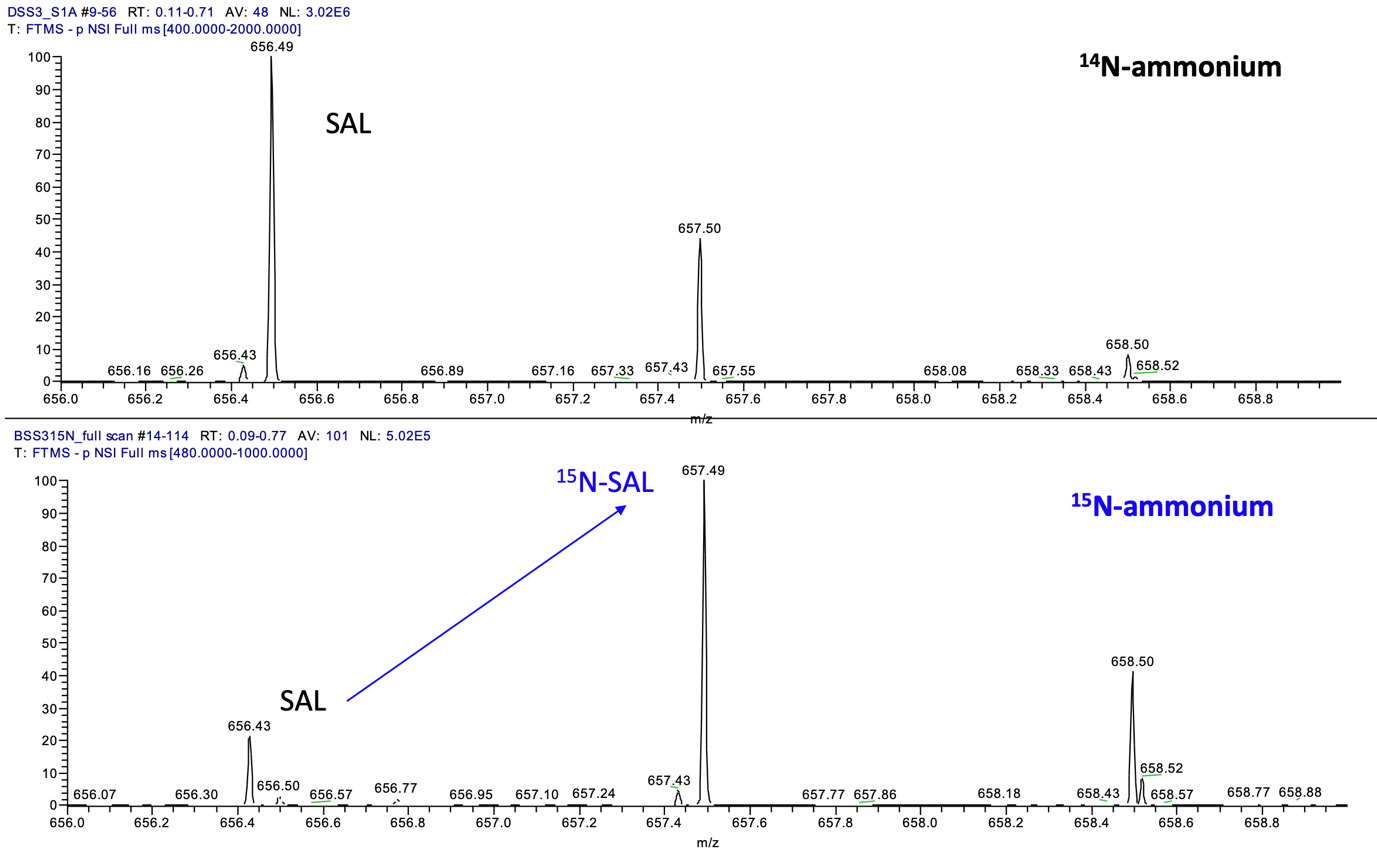


**b)**


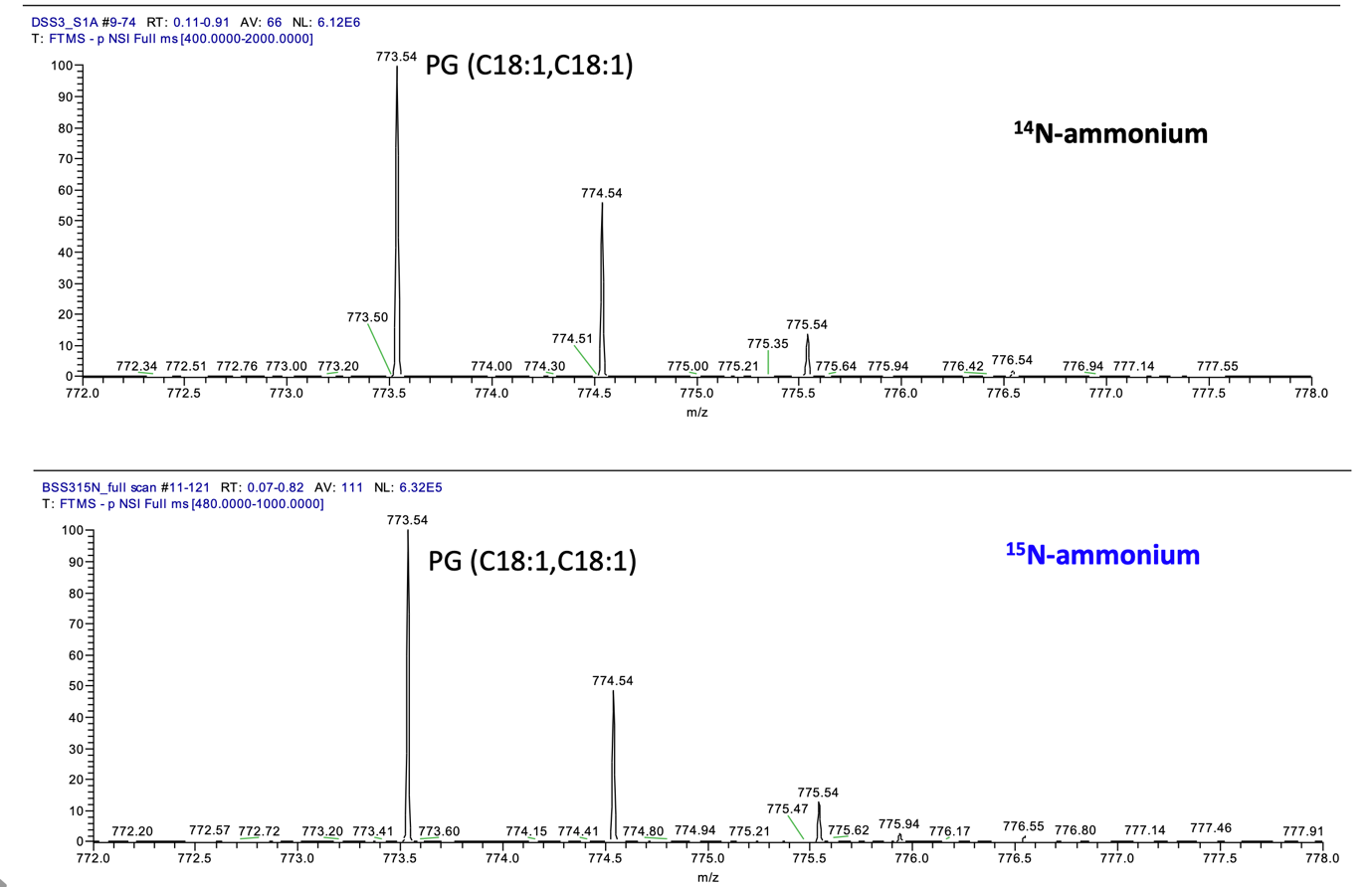


**c)**


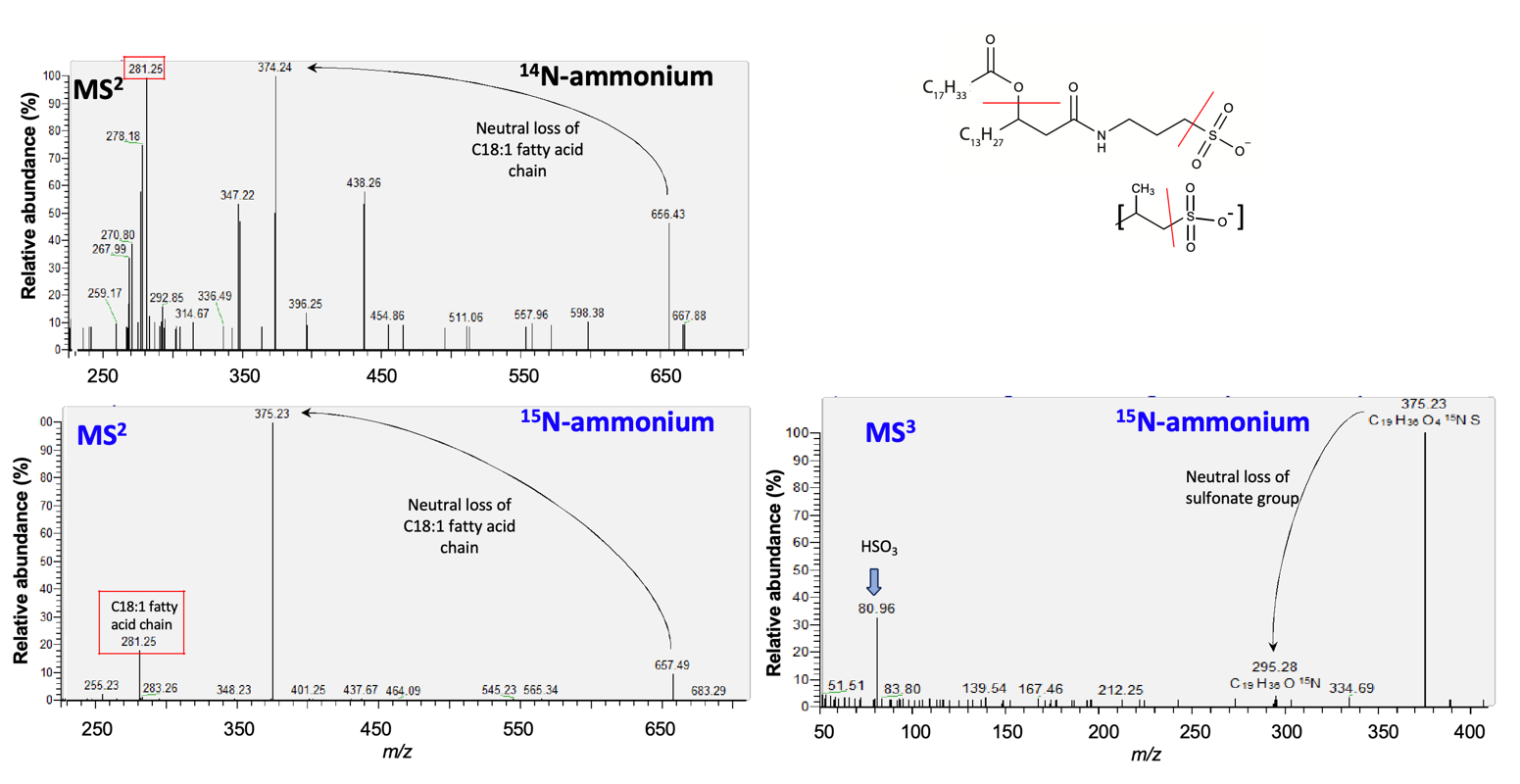


**d)**


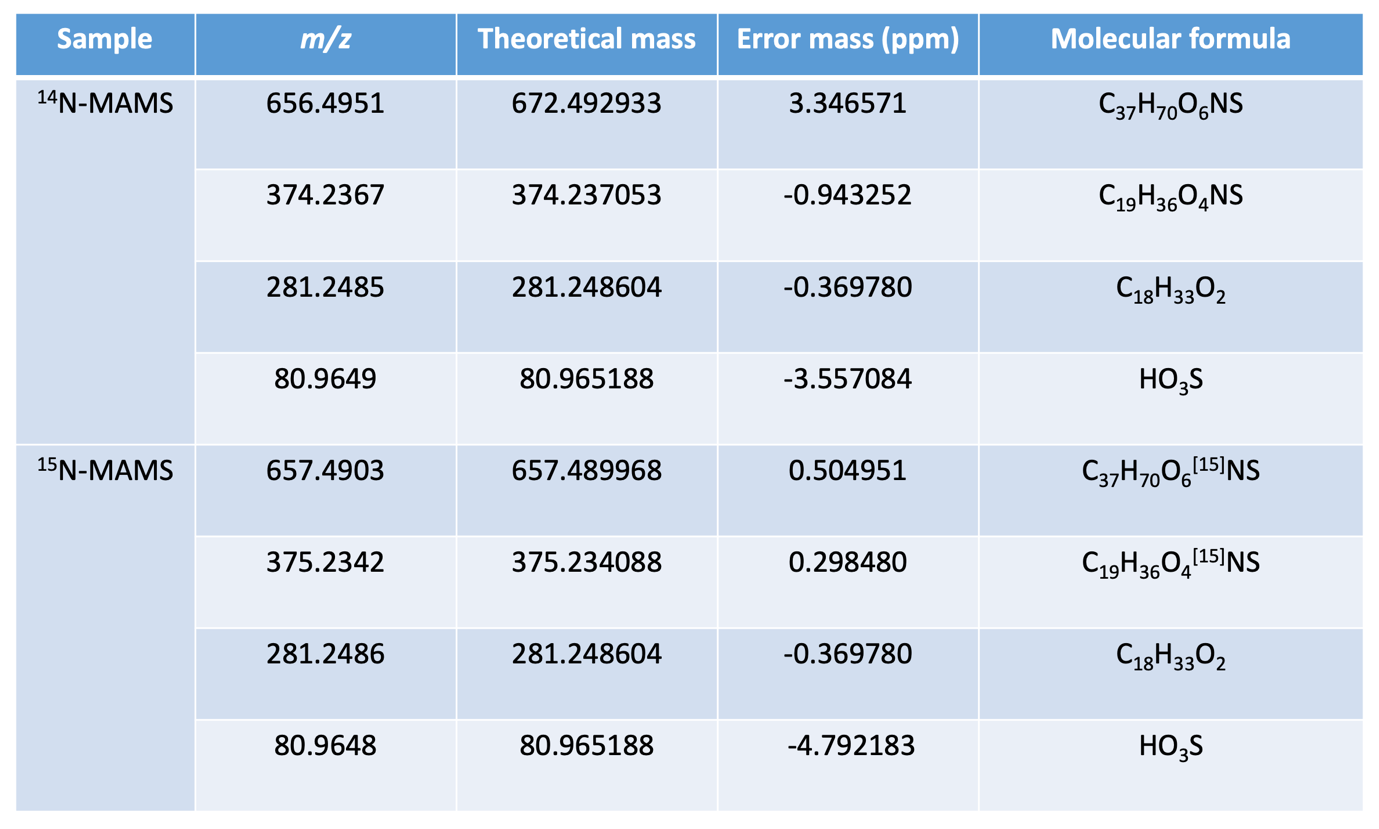


**Figure S2** **a)** *Ruegeria pomeroyi* DSS-3 cells cultivated in the defined MAMS medium supplemented with ^15^N-labelled NH_4_Cl as the sole nitrogen source resulted in a significant enrichment of ^15^N-labelled SAL (*m/z* shift from 656.49 to 657.49) in the negative ionisation mode. **b)** However, no enrichment of ^15^N in the C18:1/C18:1 phospholipid PG was observed, which does not contain nitrogen. **c)** Further MS^n^ fragmentation of the ^15^N-labelled SAL (*m/z* 657.49) showed the production of a C18:1 fatty acid (*m/z* 281.25) and a *m/z* 375.23 ion which give rise to the formation of the HSO_3_ ion (*m/z* 80.96). Ions were collected and fragmented in negative (-ve) ionisation mode using an Orbitrap fusion MS (Thermo Fisher Scientific) by direct infusion. **d)** Theoretical mass and proposed molecular formula for the identified ions from *m/z* 656 SAL species.
