# Supplementary material for: A novel class of sulfur-containing aminolipids widespread in marine roseobacters
Source: ISME J. 2021 Mar 9;15(8):2440–53. doi: 10.1038/s41396-021-00933-x (PMC8319176; doi:10.1038/s41396-021-00933-x)
Supplement: Supplementary file 4 — supplementary figure 3 [file 41396_2021_933_MOESM4_ESM.docx]

**a)**


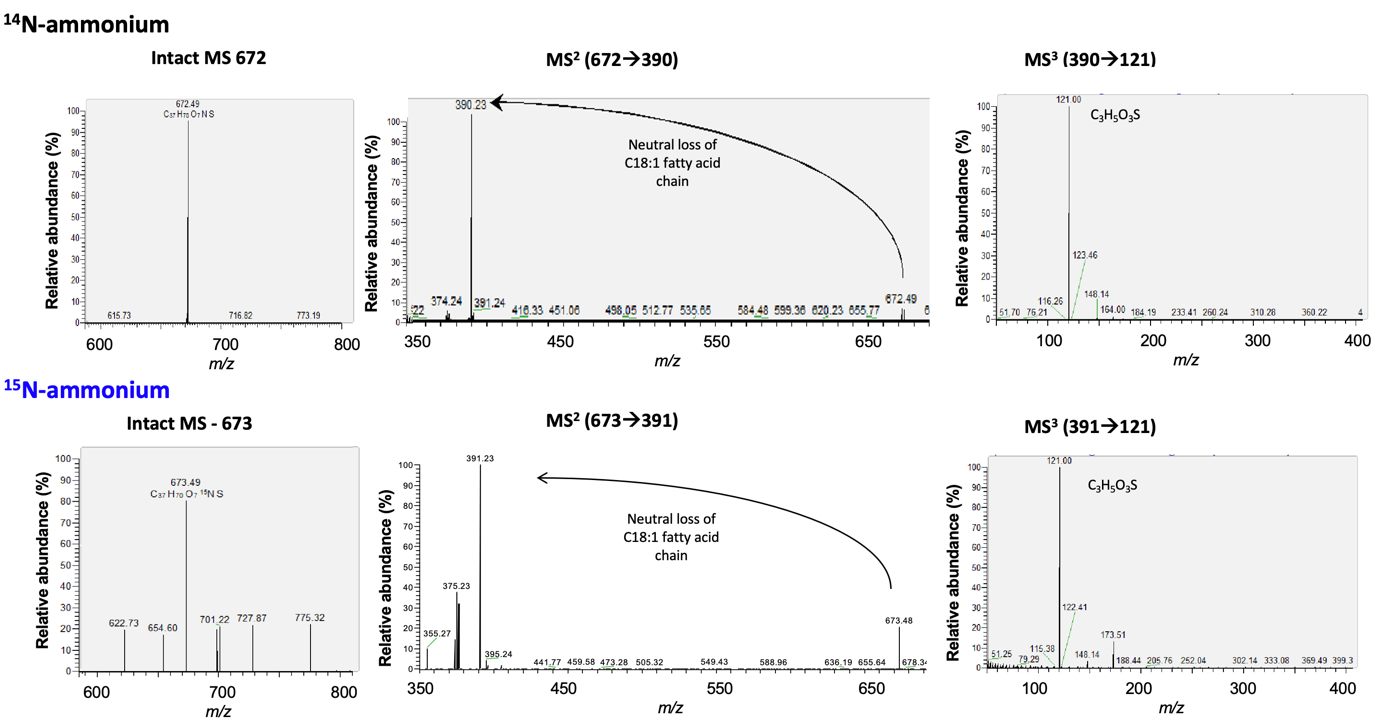


**b)**


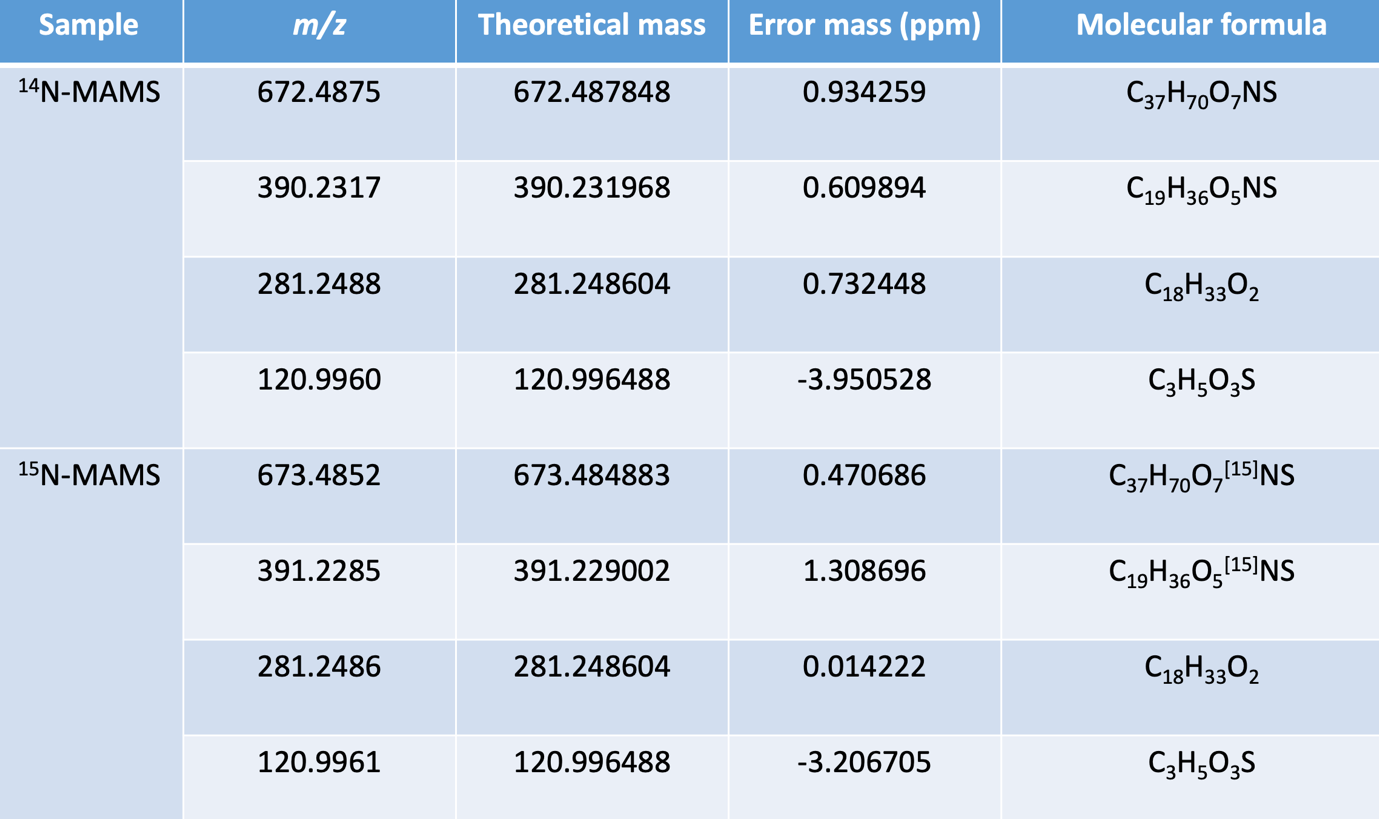


**c)**

**
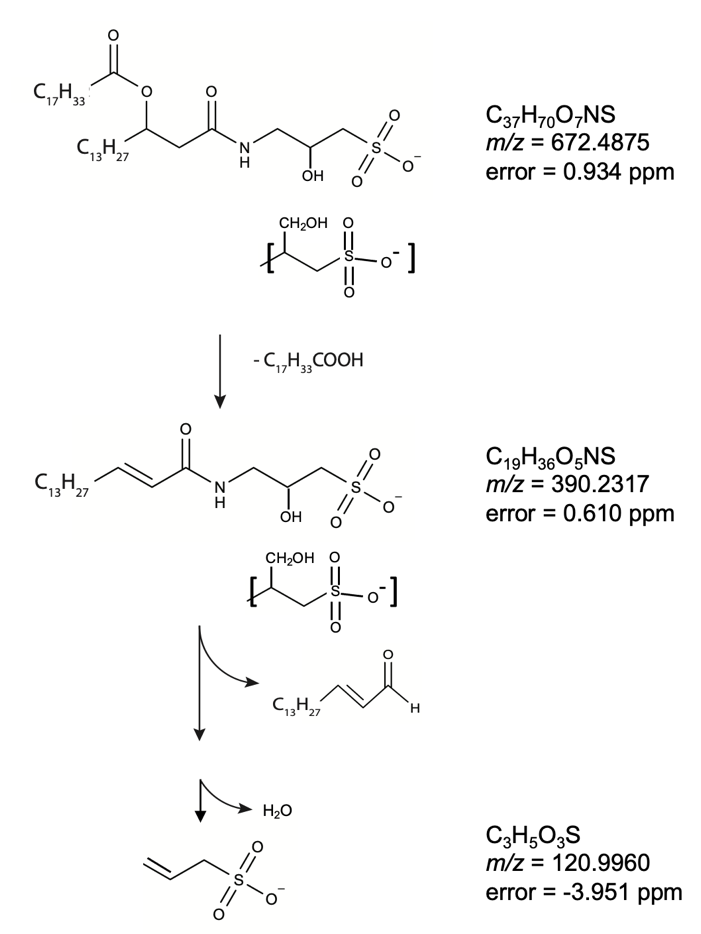
**

**Figure S3**, **a)** MS^n^ fragmentation of the *m/z* 672 and *m/z* 673 SAL lipid species extracted from *Ruegeria pomeroyi* DSS-3 cultivated in a defined MAMS medium supplemented with ^14^N-NH_4_Cl or ^15^N-NH_4_Cl as the sole nitrogen source, respectively. MS^n^ fragmentation was carried out in the negative ionisation mode using an Orbitrap fusion MS (Thermo Fisher Scientific) by direct infusion. **b)** Theoretical mass and proposed molecular formula for the identified ions are also shown. Both *m/z* 672 and *m/z* 673 SAL produced a sulfur-containing ion of *m/z* 121 (2-propene-1-sulfonate) and neither HSO_3_ ion (*m/z* 80.96) nor SO_3_ ion (*m/z* 79.96) was observed. **c)** Proposed fragmentation pattern of the *m/z* 672 SAL species.
