# Supplementary material for: A novel class of sulfur-containing aminolipids widespread in marine roseobacters
Source: ISME J. 2021 Mar 9;15(8):2440–53. doi: 10.1038/s41396-021-00933-x (PMC8319176; doi:10.1038/s41396-021-00933-x)
Supplement: Supplementary file 5 — supplementary figure 4 [file 41396_2021_933_MOESM5_ESM.docx]

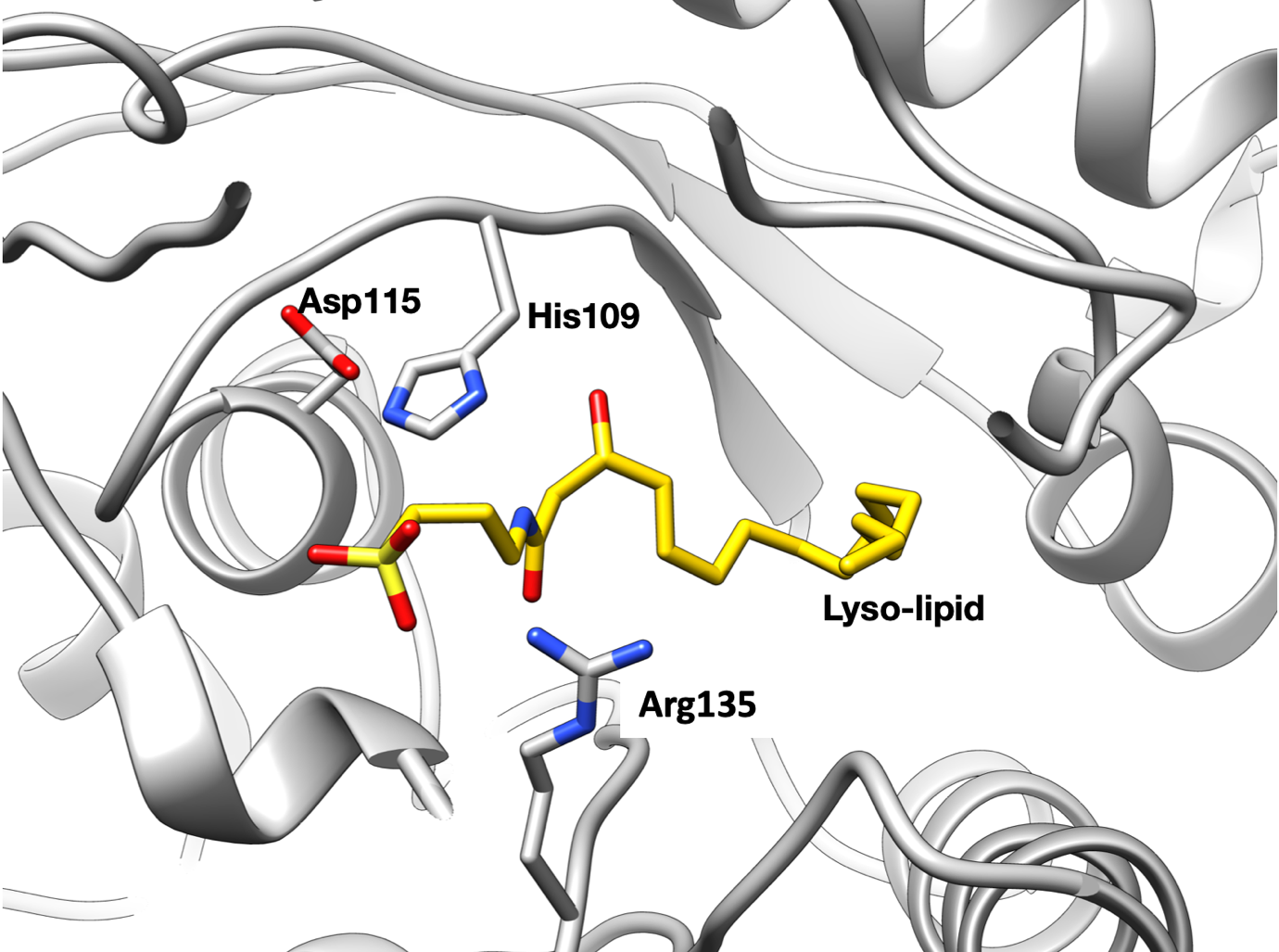


**Figure S4**, Homology model of SalA docked with the lyso-SAL lipid, showing a possible pose for the lyso-SAL lipid hydroxy group adjacent to His109 with the Arg135 suggested to coordinate the sulfonate head group.
