# Supplementary material for: A novel class of sulfur-containing aminolipids widespread in marine roseobacters
Source: ISME J. 2021 Mar 9;15(8):2440–53. doi: 10.1038/s41396-021-00933-x (PMC8319176; doi:10.1038/s41396-021-00933-x)
Supplement: Supplementary file 6 — supplementary figure 5 [file 41396_2021_933_MOESM6_ESM.docx]

**a)**

**
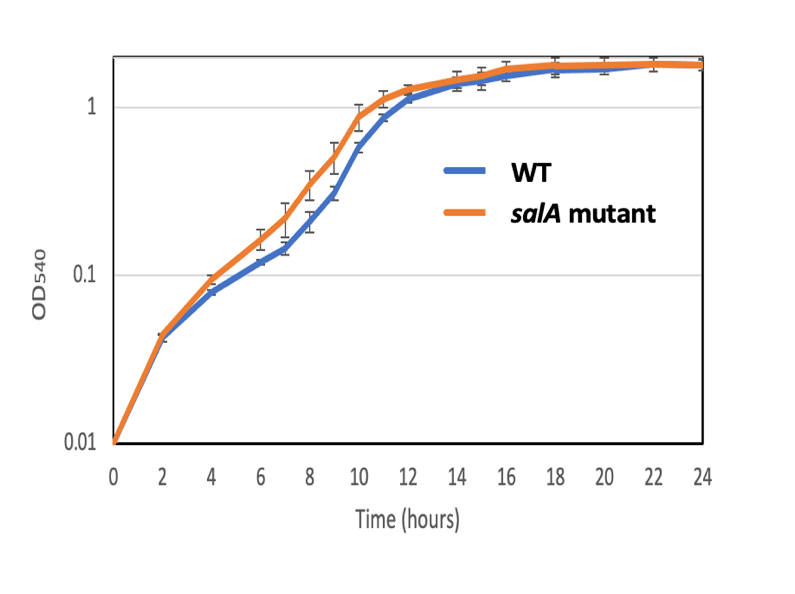
**

**b)**

**c)
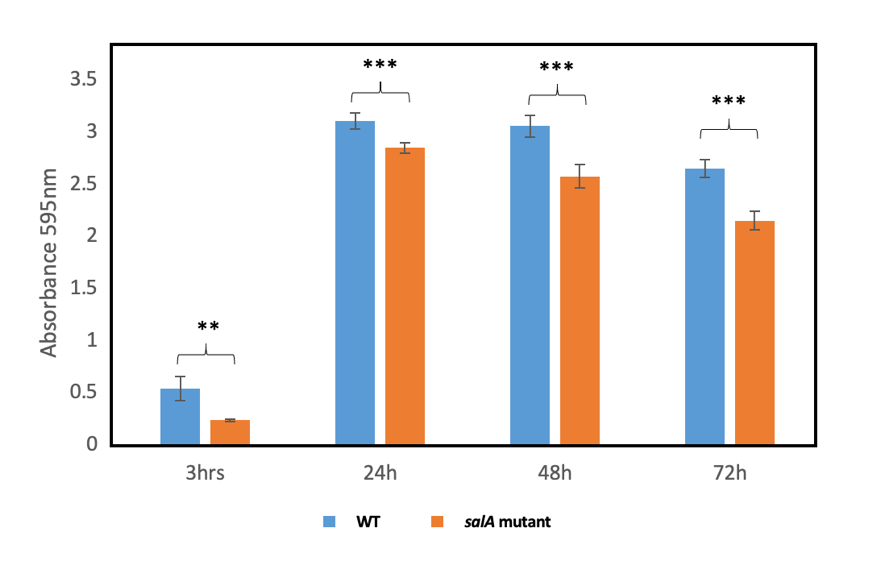
**


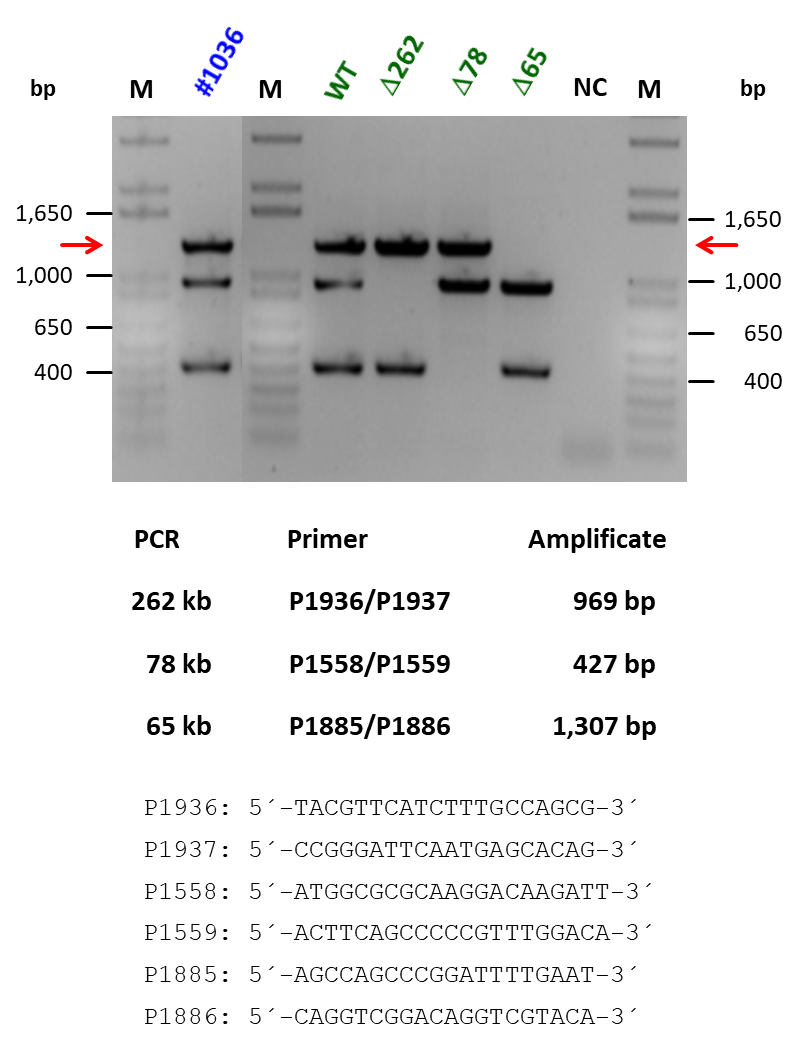


**Figure S5** **a)** Growth of the wild-type and the *salA* mutant strain of *Phaeobacter inhibens* DSM 17395 in marine broth medium. **b)** Biofilm assay showing the differences in biofilm formation on plastic surfaces in marine broth medium between the wild-type and the *salA* mutant strain of *Phaeobacter inhibens* DSM 17395. The absorbance of crystal violet was measured at 595 nm after 3 h, 24 h, 48 h and 72 h post inoculation. **, *p*<0.01; ***, *p*<0.001. **c)** Plasmid profiling of the *salA* mutant [no #1036] from *Phaeobacter inhibens* DSM 17395. Triplex PCR for the presence of the 262 kb, 78 kb and 65 kb plasmids. The wild type (WT) and three plasmid curing mutants (Δ262, Δ78, Δ65) served as a reference. Red arrows indicate the 1,307 bp PCR product of the 65 kb biofilm plasmid. NC, negative control; M, marker (Gibco, 1 Kb Plus DNA Ladder).
