# Supplementary material for: A novel class of sulfur-containing aminolipids widespread in marine roseobacters
Source: ISME J. 2021 Mar 9;15(8):2440–53. doi: 10.1038/s41396-021-00933-x (PMC8319176; doi:10.1038/s41396-021-00933-x)
Supplement: Supplementary file 7 — supplementary figure 6 [file 41396_2021_933_MOESM7_ESM.docx]

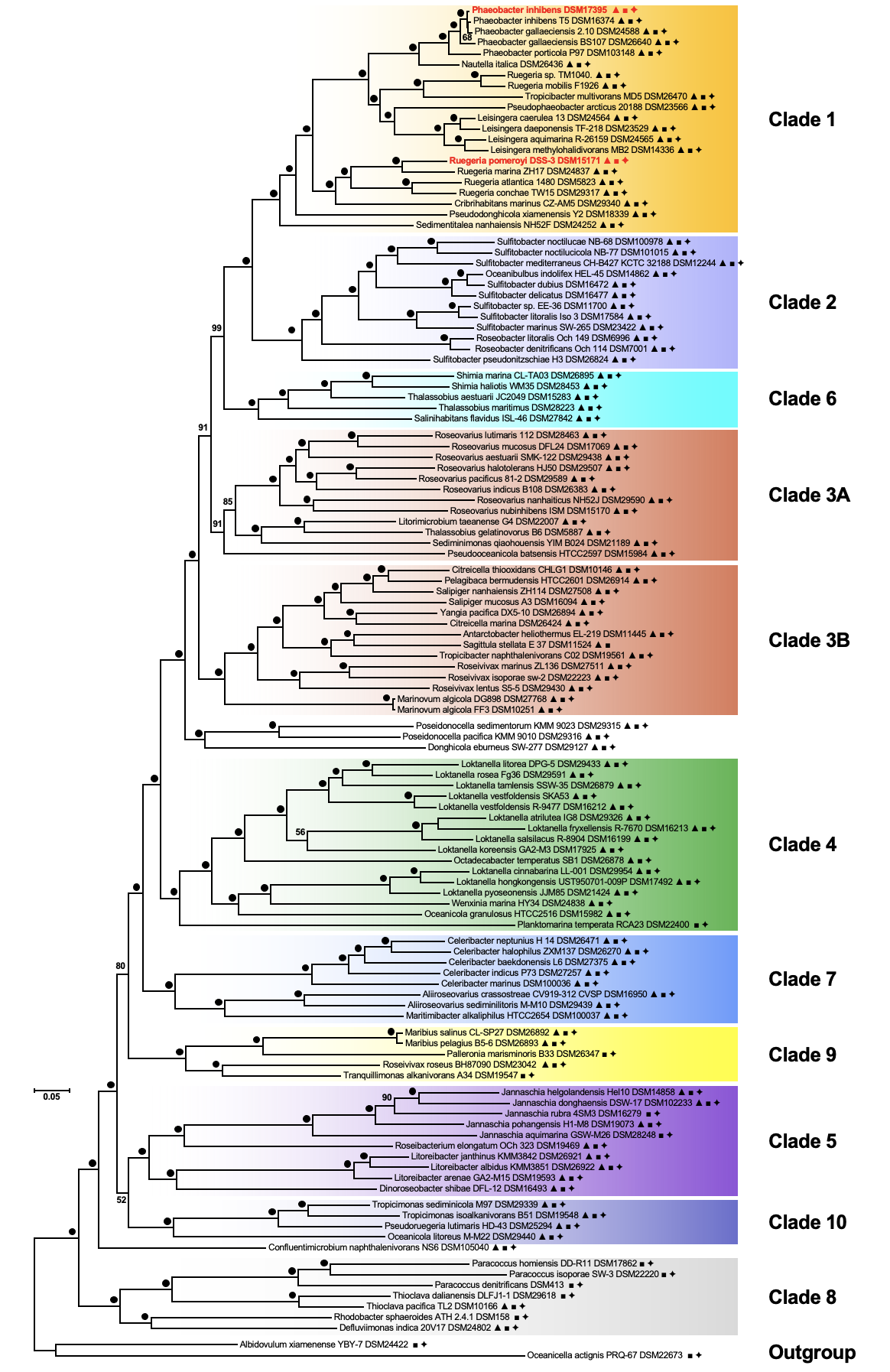


**Figure S6** Distribution of *salA* (▲), *olsA* (■) and *plsC* (✦) in genome sequenced roseobacters. The two model bacteria *Ruegeria pomeroyi* DSS-3 and *Phaeobacter inhibens* DSM17395 used in this study are highlighted in red. This phylogenomics tree was constructed by Bartling et al. (2018) using 120 genome-sequenced *Rhodobacteraceae* isolates with 504 universal marker gene alignments and a combined length of 153,625 conserved amino acid residues. Ten different lineages (Clade 1 to 10) were shown and the presence/absence of genes involved in sulfur-containing animolipid (*salA*), ornithine-lipid (*olsA*) and phosphatidic acid (*plsC*) synthesis is marked.
